# Health Tract, No. 106

**Published:** 1862-09

**Authors:** 


HEALTH TRACT, No. 106.

### BEAUTY AND MEDICINE,

NEVER before prescribed in any book or newspaper or magazine, but known in the silent experiences of millions to be almost miraculously reviving, and which, if unexpectedly "exhibited," (the officinal expression for giving a dose of medicine,) would be more instantaneously and safely efficient in the dreary hospitals and barracks, where so many of our poor soldiers, brave and patriotic, are languishing day after day, by waking up the sinking or exhausted powers, than all the pills and potions in the universe. In its good effects it is infallible, perfectly safe, and always unmistakably agreeable; it thrills the whole man, physical and moral, with delight. In one respect it is curiously different from any other medicine. A single pill, potion, or powder of the apothecary serves only for that once and for a single individual, but this new medicine, which, as far as we know, HALL'S JOURNAL OF HEALTH is the first to bring definitely into public notice, goes a great way; it is capable of infinite (not "infinitesimal") extension; it goes further, by a million times, than the Hahnemann's millionth "dilution." The same potion will benefit each of a thousand sick soldiers as much as it will benefit one; and that identical potion can be used by any other thousand soldiers, and other continuous thousands, with the same happifying results. The greatest drawback is, that it is not the most plentiful thing in the world; still, it is more or less abundant in any five miles square of cultivated soil. When the hospital-surgeon takes his regular daily morning rounds, let him be followed by

A pretty girl with laughing curls.

That is all, reader; and it is a sober truth, every word of it. But to make a more specific and practical application, let half a dozen girls, tidily dressed, with countenances beaming with youth and gladness and genial sympathy, pass through the wards of any hospital of soldiers at a regular hour every morning, each having on her arm a large basket of *small* bouquets, so as to go the further, and each followed by a servant with a hamper filled with baskets of berries; a basket or a bouquet to be handed to each patient by the sweet visitors, accompanied with a single, kind, sympathizing or encouraging word. The unexpected sight of a beautiful little flower in the desert, waked up into courageous and life-saving effort the desponding and dying Mungo Park. A ship's crew, cast away on the bleak and frozen shores of the Arctic sea, dug out of the dreary snow-bank the handle of an old pewter spoon, and on turning it over and seeing the name of "London" on it, which they had left three years ago, they burst into tears, and with these came new resolves to brave all dangers until danger should be past, which they did. The sight of an old bonnet, in the early days of California, unexpectedly come upon by a company of sad and weary and disappointed miners, so roused their sinking spirits, that with a yell and a hurrah they formed a ring, and with uproarious songs, danced around the dilapidated remnant of a higher civilization. Within a week, a gifted lady presented a clean muslin handkerchief to a sick soldier in the hospital. With his trembling fingers he spread it out before him, and buried his face in its folds in silent, long, and inexpressible delight. When one of the public schools sent a cart-load of bouquets, deputing a dozen or more of the prettiest scholars to distribute them among the sick soldiers in the Park barracks, a month ago, the effect was so electrical and overpowering on the men, expressed in various ways, that some of the bystanders could not refrain from tears. And all this because both the flower and its bearer carry the mind back to home, its sweetness, its purity, its affection, and its sunshine, and wakes up a new ambition for life and a determination to live down the present sorrow, so as to drink in again the joys and the sunshine of home once more. These are sober facts, scientific facts, founded in human nature, and they ought to be made use of. Who does not know that such a visitor, expected at a given hour at any hospital, would be an event to be looked for with pleasure, and would be prepared for by greater and greater attempts to make and improve the toilet more and more early in the day? An influence would go out more efficacious than any exhortation or command or threat in promoting tidiness and cleanliness on the part of the soldier himself. It may seem a trifling joke, but it is a sober reality, and could not possibly fail of a largely beneficial application. Why, a dying patient was once waked up into life from the fatal lethargy of typhoid by the sudden and unexpected entrance of an old sweetheart. Beauty is a power; we feel it in our bones every time we come in sight of it; and we verily believe that the older we get the worse we are in this regard. Wonder if it is so with other young men of our age?

